# Breaking Barriers in Huntington’s Disease Therapy: Focused Ultrasound for Targeted Drug Delivery

**DOI:** 10.1007/s11064-024-04302-w

**Published:** 2025-01-03

**Authors:** Mohamed Mohsen Helal, Arwa Amer Ibrahim, Ahmad Beddor, Muataz Kashbour

**Affiliations:** 1 Faculty of Medicine, Zagazig University, Zagazig, Egypt; 2 Medical Research Group of Egypt, Negida Academy, Arlington, MA USA; 3 College of Medicine, University of Sharjah, Sharjah, United Arab Emirates; 4 Faculty of Medicine, Yarmouk University, Irbid, Jordan; 5 Diagnostic Radiology Department, National Cancer Institute, Misrata, Libya

**Keywords:** Huntington's disease, Focused ultrasound, Neurodegenerative diseases, Blood-brian barrier, Drug delivery, Clinical trials

## Abstract

Huntington’s disease (HD) is a progressive neurodegenerative disease resulting from a mutation in the huntingtin (HTT) gene and characterized by progressive motor dysfunction, cognitive decline, and psychiatric disturbances. Currently, no disease-modifying treatments are available. Recent research has developed therapeutic agents that may have the potential to directly target the disease pathology, such as gene silencing or clearing the mutant protein. However, these agents are limited by their inability to cross the blood-brain barrier (BBB), preventing optimal therapeutic effects. Although various techniques have been explored to overcome the BBB, focused ultrasound (FUS) has emerged as a promising non-invasive therapeutic modality offering the potential for targeted intervention in neurodegenerative diseases, including HD. Preclinical studies demonstrated the safety and efficacy of FUS in delivering therapeutic agents, such as siRNAs and AAV vector-based gene therapy, resulting in significant reductions in mutant HTT expression and improvements in motor function in HD mouse models. Furthermore, the safety profile of FUS-induced BBB opening has been established in clinical trials on human patients of neurodegenerative diseases other than HD, showing no adverse effects on brain structure or function. This review provides a comprehensive overview of the current state of FUS research in HD and connects existing evidence from neurodegenerative disease studies with its promise in establishing disease-modifying therapies for HD.

**Graphical Abstract:**

**Figure:**
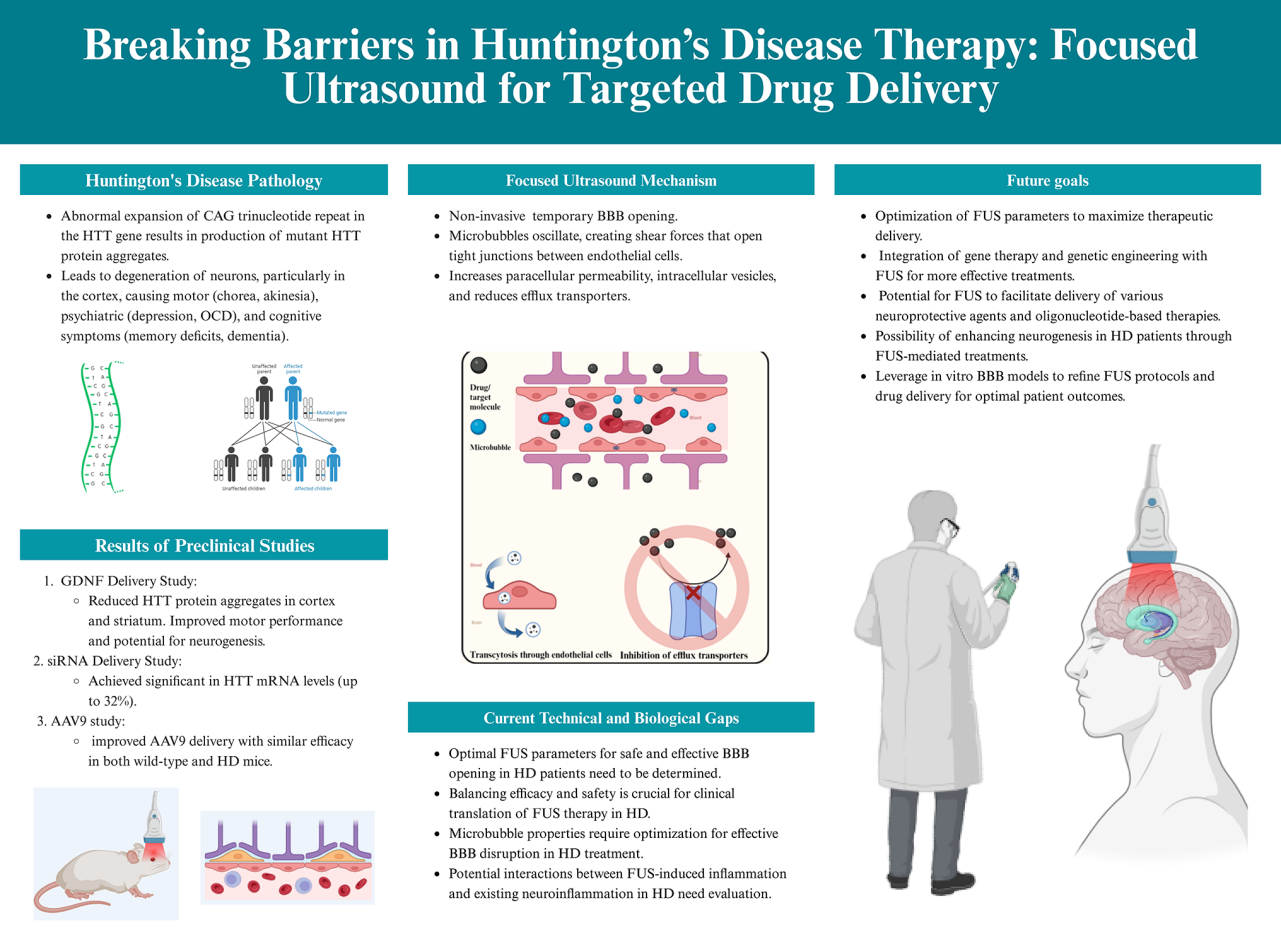
Created with BioRender [1]

## Introduction

Huntington’s disease (HD) is a genetic neurodegenerative disorder affecting more than 30,000 people in the US and putting more than 200,000 at risk of its inheritance [2]. The culprit genetic mutation is an abnormal expansion of the trinucleotide repeat CAG (cytosine-adenine-guanine) in the huntingtin (HTT) gene (IT-15 previously) found on the short arm (p) of chromosome 4 [3]. This mutation results in the production of the HTT protein with an abnormally high number of glutamine amino acids (polyglutamine-expanded aggregates) [3]. These cellular-based changes result in the degeneration of neurons, particularly the cerebral cortex and basal ganglia. Therefore, HD leads to a variety of motor, psychological, and cognitive symptoms.

HD typically begins in middle age and progresses over time. The main motor symptom of HD is chorea [4, 5], which is substituted by akinesia with the progression of the disease. In addition, many psychiatric manifestations are documented to accompany HD, like bipolar disorders and obsessive-compulsive disorder, and the most commonly associated one is depression [3]. Its devastating psychological effect extends to patients’ suicidal attempts. A cohort study of HD patients revealed that suicide forms 3–7% of HD-related deaths [6]. Furthermore, its cognitive symptoms primarily involve deficits in memory, speech, vision, processing, attention, and other cognitive functions [4, 5, 7, 8]. Moreover, dementia usually develops, making HD patients fully dependent on caregivers for their everyday activities [9]. Now, HD dementia is considered a differential diagnosis of dementia with Alzheimer’s disease (AD) and Parkinson’s disease (PD) dementias and has full criteria to be diagnosed [3].

HD is transmitted in an autosomal dominant manner and exhibits *complete penetrance*, ensuring that every person with the mutation will develop the disease [3]. A key feature of HD is the instability of the CAG trinucleotide repeats within the HTT gene, which tend to expand in size with each generation, leading to a phenomenon known as genetic anticipation. This phenomenon results in an earlier and more severe presentation of HD in subsequent generations, making it increasingly challenging to manage. Addressing this progressive nature of HD necessitates the development of effective treatments to interrupt the disease cycle.

Recent advances in medical research have significantly improved our understanding of the role of the HTT gene mutation in the pathology of the disease. Several theories have been adopted, including mitochondrial dysfunction, proteolysis by caspases and other proteases, and excitotoxicity [10–12]. The first one supposes that the mutant HTT (mHTT) leads to mitochondrial dysfunction as a result of impaired calcium regulation and reduced adenosine triphosphate (ATP) production, which finally causes neuronal cell apoptosis [13]. The proteolysis theory suggests that cytotoxicity and neurodegeneration result from the cleavage of HTT protein in the cytoplasm and translocation of the fragments into the nucleus [11]. These theories have set the stage for potential therapeutic agents that could compensate for such changes. For instance, creatine and coenzyme Q10 are being explored to address mitochondrial dysfunction as they act as a source of ATP, caspase-6 inhibitors for proteolysis, and riluzole for excitotoxicity [13]. Additionally, stem cell therapies are being investigated to address neuronal cell loss.

Nevertheless, given the identified primary pathology underpinning HD, which is the mHTT, various HTT-lowering strategies are being developed. Such strategies focus on disrupting the DNA or RNA of mHTT or preventing the aggregation of the abnormal HTT protein in neuronal cytoplasm. Among these, interfering RNA of the HTT gene is the most advanced strategy for this purpose, having been tested in phase I, II, and III clinical trials [14–17]. RNA interference is implemented through a variety of available methods, including, but not limited to, antisense oligonucleotides (ASOs), which are widely used, single-stranded RNA molecules (ssRNAs), and artificial microRNAs (miRNAs) [18, 19]. ASOs exert their silencing action by binding to the HTT mRNA and interrupting the biosynthesis of the protein [3]. Other RNA interference strategies have a mechanism of action similar to that of ASOs [20]. Additionally, disrupting DNA has been proposed as a potentially effective approach. By directly targeting the mutant gene (and reducing the number of CAG repeats), it could delay disease onset and alleviate symptoms [20]. However, this strategy has not yet progressed beyond preclinical studies.

A significant debate surrounding the aforementioned HTT knockdown strategies stems from the recognized physiological functions of the HTT gene and protein in human brain development, neuronal survival, protein synthesis, and impulse transmission [21]. However, the complete physiological role of HTT remains unclear. To address this, various preclinical trials investigating the effects of HTT silencing have been conducted, yielding mixed results [22, 23]. Consequently, the potential negative consequences of implementing such strategies in humans are still uncertain [24].

Currently, there are no approved disease-modifying treatments for HD [10, 25, 26]. The available therapies mainly focus on symptomatic management. Chorea is typically managed with presynaptic dopamine-depleting agents such as tetrabenazine [3]. Depression is often treated by selective serotonin reuptake inhibitors (SSRIs) like fluoxetine, and dementia is treated by cholinesterase inhibitors [3].

One of the primary *challenges* in treating neurodegenerative diseases, such as HD, is the inability to deliver therapeutic agents across the *blood-brain barrier (BBB)*. The BBB serves as a protective layer that shields the brain from harmful substances but also restricts the penetration of many drugs [27]. In certain cases, the BBB can significantly impede the delivery of therapeutic agents, preventing them from reaching effective concentrations in the brain [28]. This limitation has hindered the clinical application of numerous compounds that have demonstrated efficacy in treating brain diseases during animal studies by direct delivery methods.

For a drug to be suitable for clinical use, it must effectively penetrate BBB while being administered through a *non-invasive* route. This requirement is essential to ensure that therapeutic agents can reach the brain safely and efficiently, minimizing the risks associated with invasive procedures.

In HD research, therapies are continually evolving to address the various theories of disease pathogenesis. For instance, small interfering RNA (siRNA), which is one of RNA interference methods, has shown a favorable safety profile and efficacy in reducing levels of mHTT in non-human primates and mouse models following direct injections into the brain parenchyma or ventricles [29, 30]. However, it must be delivered in a less invasive manner and successfully cross the BBB to achieve adequate brain concentrations to be a viable treatment option for humans with HD. Research using various materials and methods is exploring the most effective approach to achieve this goal [31, 32].

Among the techniques used to overcome the BBB, one promising and emerging strategy is *Focused Ultrasound (FUS)*. FUS has demonstrated its potential as a non-invasive and targeted brain delivery method [33]. This is particularly beneficial in neurodegenerative diseases since more localized treatment may be more effective. FUS has enhanced the effectiveness of various HD therapies, such as siRNA, glial cell line-derived neurotrophic factor (GDNF), and adeno-associated virus (AAV) vector-based gene therapies, through effective BBB opening in animal models. This approach could revolutionize the treatment of HD disease. Although FUS-mediated treatments of neurodegenerative diseases are in various stages of basic, preclinical, and clinical research, they offer hope that HD may one day be treatable [34, 35]. However, many challenges remain before these therapies become available alternatives. Here, we provide a comprehensive review of FUS-mediated BBB opening in HD. We also highlight the promising potential and future directions for managing HD using FUS.

## Crossing the BBB: Strategies for Medication Delivery and Associated Challenges

The BBB is a semipermeable, multicellular structure within the brain’s capillaries that encloses and protects the brain from harmful substances such as toxins, certain drugs, and microorganisms [27]. It plays a crucial role in regulating the brain’s microenvironment and maintaining homeostasis [36–38]. Due to its highly selective permeability, it represents one of the most resistant barriers in the body [39].

The primary structural unit of the BBB is the *neurovascular unit (NVU)* [27], which consists of specialized endothelial cells (ECs), pericytes, astrocytes, adjacent neurons, and the basement membrane. These components work together to maintain the integrity and function of the BBB. The ECs are interconnected by tight junctions (TJs) and adherens junctions (AJs), which limit paracellular permeability. Unlike peripheral capillaries, the ECs of the BBB lack fenestrations, further restricting transcellular permeability [40]. Moreover, these ECs contain significantly fewer vesicles, which limit intracellular transcytosis, and they are equipped with numerous efflux transporters, such as P-glycoprotein, which actively pump drugs out of the brain [41].

Collectively, the NVU forms an extensive network that encloses approximately 600 km of capillaries within the human brain [42]. This extensive barrier creates a significant challenge in the delivery of neurotherapeutic agents, effectively blocking 98% of small-molecule neurological drugs and nearly 100% of larger ones [28].

Research has been focused on overcoming the BBB’s selective permeability and the associated challenges in delivering neurotherapeutics since the late 20th century. Applying hyperosmotic solutions, e.g., mannitol and urea, was commonly used in the first attempts at BBB opening [33, 43, 44]. These solutions cause cellular shrinkage, which leads to the loosening of the TJs between ECs, thereby increasing paracellular permeability [45]. However, this method requires invasive intra-arterial catheterization and produces off-target openings in the territory of the injected vessel [46, 47].

To overcome the diffuse effect, researchers tried direct injection of the drugs into the target brain region through the skull [47]. Although this approach to overcoming the BBB is more targeted, it is highly invasive and carries significant risks, including infection, bleeding, and tissue damage. To address these limitations, non-invasive techniques such as FUS have emerged, offering a controllable method to temporarily disrupt the BBB for drug delivery without the need for invasive procedures [33].

### Mechanisms and Evolution of FUS for BBB Opening

FUS has undergone significant advancements, demonstrating its effectiveness in non-invasively opening the BBB while overcoming the limitations associated with other techniques [48]. One of the key advantages of FUS is its ability to create *localized* openings in the BBB without causing damage to surrounding neurons [48]. Notably, a pioneering study revealed that FUS-mediated BBB openings are transient, allowing the barrier to heal and restore its normal function and structure following sonication [49]. This unique characteristic of FUS, along with others, not only minimizes potential risks but also underscores its promise in the treatment of chronic brain diseases that necessitate long-term drug administration.

FUS acts through temporarily *disrupting* the internal mechanisms of the BBB and its endothelial cells (ECs). Typically, FUS is combined with the intravascular administration of microbubbles (MBs) [40]. MBs, which are gas-filled components of a contrast agent injected prior to FUS application, have physical parameters that significantly affect the resulting BBB disruption [50].

**Fig. 1:**
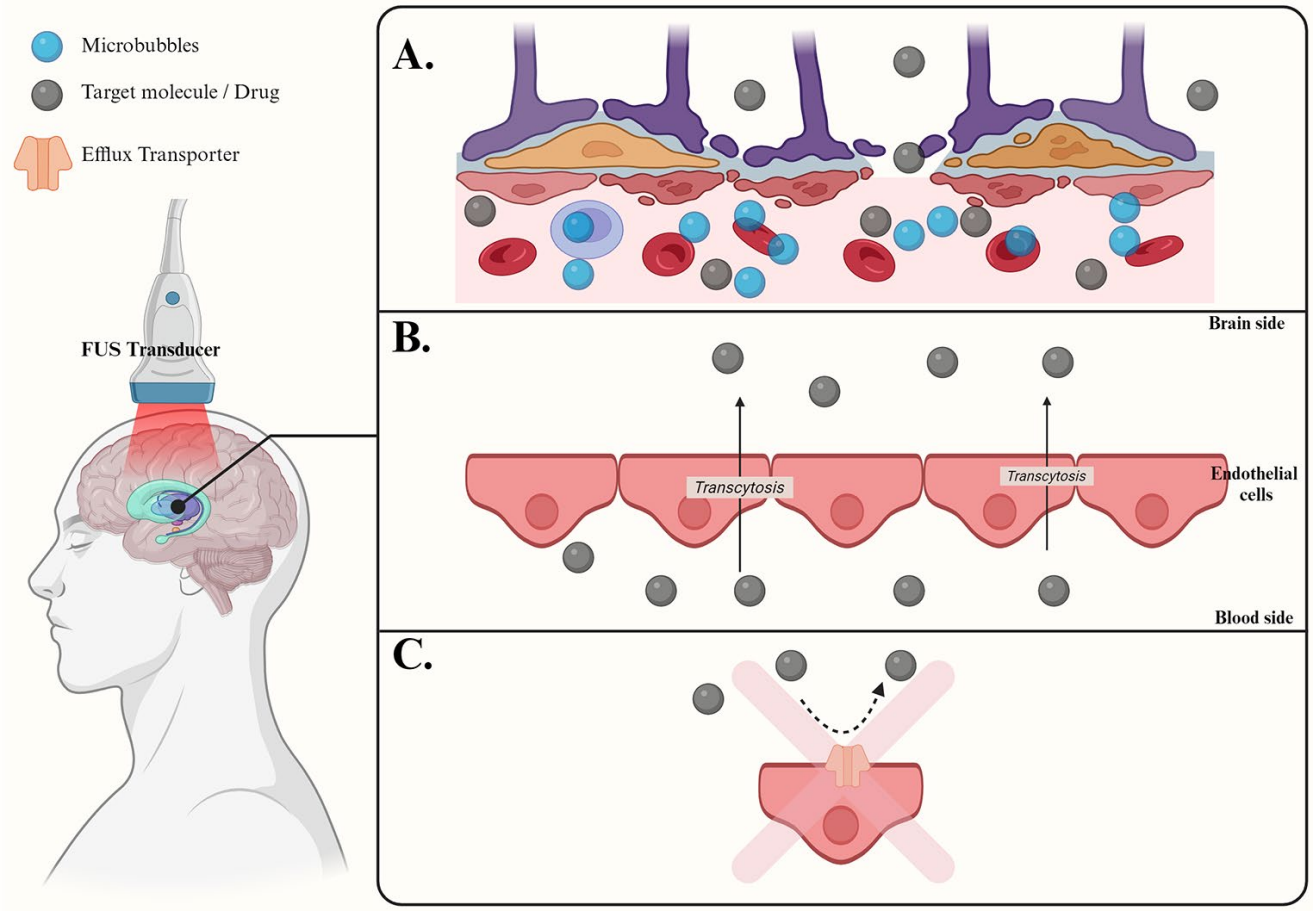
Schematic Illustration of the Mechanism of FUS-Induced BBB Opening: This figure illustrates how focused ultrasound (FUS) combined with microbubbles (blue) enhances drug delivery across the blood-brain barrier (BBB). In Panel A, FUS is applied externally to the head, causing the injected microbubbles in the bloodstream to oscillate and temporarily disrupt the BBB, allowing drug molecules (gray) to enter the brain. Panel B shows that FUS increases transcytosis across endothelial cells, facilitating drug transport from the blood to the brain. Finally, Panel C demonstrates that FUS can inhibit efflux transporters, which usually pump drugs out of the brain, enabling drugs to stay in the brain longer. Figure created with BioRender [1]

Upon sonication of the target region, the MBs undergo rhythmic contraction and expansion, generating shear forces on the blood vessels in this area—a phenomenon known as the “cavitation effect” [51]. This physical interplay between the ultrasound beam, MBs, and the vessel walls temporarily opens the TJs between the ECs of the BBB, increasing paracellular permeability and facilitating drug delivery for a limited duration (Fig. 1).

Further research into the mechanisms by which FUS produces BBB disruption has revealed additional effects of FUS combined with MBs that impact nearly every aspect of ECs functions. Studies have demonstrated that FUS + MB increases the number of intracellular vesicles, thereby promoting transcytosis within BBB ECs [52]. Additionally, exposure to FUS + MB is believed to decrease the number of efflux transporters, which limits the removal of therapeutic agents from the brain and enhances their therapeutic effects [35, 36].

Furthermore, FUS combined with microbubbles offers a wide range of options for modulating its effects on the BBB and surrounding tissue. By calibrating ultrasound parameters such as pulse repetition frequency (PRF), pressure, and burst duration, as well as adjusting MBs characteristics like the type, dose concentration, distribution, and half-life, researchers can tailor the technique according to skull thickness, target depth, and the desired degree of effect [51, 53].

Numerous preclinical studies have aimed to optimize the parameters of FUS and MBs for maximal safety and efficacy in BBB opening by exploring the effect of changes in each parameter [51, 54, 55]. One study applied FUS + MB with varying parameters across 100 different non-overlapping brain regions in rabbits, finding that FUS burst length was the only parameter that significantly altered the extent of BBB disruption, while MBs dose and PRF had no effect [51]. In contrast, Shin et al. concluded from their optimization trial on rats that the MBs dose and the PRF could affect the magnitude of BBB disruption [54]. Species differences in skull thickness and brain volume can affect acoustic attenuation, potentially limiting the translation of FUS to clinical trials due to a lack of optimization. However, extensive research has established key findings regarding FUS + MB characteristics for human-tolerated schemes; for instance, acoustic pressure and MBs diameter are directly related to the degree of BBB opening [40]. The clinical relevance of these findings is substantial. For example, a specific range of acoustic pressure (0.2–1.0 MPa) is utilized in clinical trials for FUS-mediated BBB opening [56, 57].

Moreover, magnetic resonance imaging-guided FUS (MRgFUS) has further enhanced the technique by enabling *real-time monitoring* of the drug delivery process and intraoperative feedback. This is possible through real-time MR thermography and acoustic spectrum monitoring (size and site of the target) [58, 59]. In addition, evaluation of the effects of changing certain FUS or MBs parameters was possible using MRI through quantification of the barrier interruption and drug penetration [51, 60].

It is important to note that the FUS technology discussed in this review specifically refers to *low-intensity FUS (LIFUS)*, which is applied in BBB opening trials. In contrast, high-intensity FUS (HIFUS) utilizes high-energy ultrasonic waves for thermal ablation of tissues or neuromodulation and is currently approved for the treatment of some tumors, PD, and essential tremors [60, 61].

Demonstrating these valuable properties and versatility, FUS has transformed the field of BBB opening and, consequently, the treatment of neurodegenerative diseases. Currently, FUS has been under the scope of clinical research for years after showing favorable safety and efficacy profiles in BBB opening in animal models of neurodegenerative diseases. It has been applied in clinical trials of AD, PD, and amyotrophic lateral sclerosis (ALS) and proved its safety for human application (with some trials reporting no adverse events) [56, 62–64]. However, its efficacy as a drug delivery system for neurodegenerative disease patients has not been proven yet. In addition, FUS has not been tested in HD patients before. Therefore, summarizing the existing preclinical evidence regarding its application in HD models will provide insights into its potential as a drug delivery method for HD.

### FUS in HD: Preclinical Evidence

In recent years, FUS has garnered attention as a promising technique for enhancing treatments for HD by facilitating drug delivery to the brain. This innovative method has significantly increased the bioavailability of neuroprotective agents, improving therapeutic outcomes. Recent preclinical trials in mouse models have demonstrated the safety and efficacy of FUS-mediated delivery systems to enhance therapeutic outcomes by improving motor function and reducing toxic protein aggregates in HD [32, 65, 66].

### GDNF

One promising approach to HD treatment involves delivering GDNF to the affected neurons. GDNF is a neurotrophic protein encoded by the GDNF gene. It is naturally expressed in healthy brains, exerts neuroprotective effects, and promotes their survival and maintenance by signaling through GFRα1 receptors [67]. GDNF overexpression has been found to have positive neuroprotective effects in HD patients [68]. However, delivering GDNF to targeted areas of the brain is challenging because it does not efficiently cross the BBB.

Current methods for GDNF administration, such as intrastriatal or intraventricular injections, are invasive [69], which may have adverse effects on the patient’s health and may also diminish the potential therapeutic benefits. A previous study assessing the safety of intracerebroventricular catheter implantation for GDNF delivery in PD reported several adverse events, including paraesthesia, nausea, weight loss, and asymptomatic hyponatremia, without significant improvement in symptoms [69]. A case report highlighted the adverse effects experienced by a PD patient following GDNF injections, noting a lack of improvement in parkinsonism and a worsening of symptoms, including hallucinations, depression, nausea, and loss of appetite [70]. Such findings highlight the need for alternative, less invasive delivery methods.

FUS-induced BBB opening has shown promise as an alternative approach to delivering GDNF. In a study using HD mouse models, FUS was used to deliver GDNF plasmid DNA (GDNFp) into HD mice and reported positive results [65]. The animal model used in the study is a transgenic mouse model for HD, specifically the R6/2 HD mice. The R6/2 HD mice exhibit similar symptoms to human HD, including motor impairments and neuropathological features like protein aggregation, oxidative stress, and neuroinflammation. MRI and histological staining demonstrated the efficacy of FUS-induced BBB opening in mice without significant capillary or brain tissue damage. The study showed increased BBB permeability in the sonicated brains of both HD and wild-type (WT) mice and effective extravasation of GDNFp-liposomes (GDNFp-LPs) into the CNS, resulting in an increase in GDNF protein expression in the target cells.

To investigate the pathological changes, multiple immunohistochemical stainings showed significant reductions in HTT protein aggregates in the cortex and striatum of HD mice, indicating a positive therapeutic effect. Additionally, there was a significant increase in the expression of the neuronal survival marker, phosphorylated cAMP response element-binding protein (P-CREB), and improved motor performance in the treated HD mice. Elevated levels of microtubule-associated protein 2 (MAP2) suggested enhanced neurite outgrowth. As a result, neurogenesis was found to be a potential outcome of the GDNF treatment, further supporting the efficacy of this non-invasive FUS-mediated BBB opening technique for gene therapy in addressing the neurodegenerative processes associated with HD.

The promising preliminary findings of this study lead future research to focus on modifying FUS parameters to maximize GDNF delivery, evaluating the long-term outcomes in larger animal models, and transitioning to trials on humans. This method not only improves neuronal survival and neuroprotection but also supports neurogenesis, which is critical for tackling the neurodegenerative aspects of HD. Additionally, gene therapy and genetic engineering integration to utilize more effective delivery methods can pave the way for breakthroughs in HD treatments.

### Oligonucleotide-Based Therapies

Given that the mHTT protein is the primary cause of HD, oligonucleotide-based therapies using siRNAs and antisense oligonucleotides, designed to specifically silence mHTT, hold promise as novel treatments [71]. siRNAs are small, double-stranded RNA molecules that can degrade mRNA, preventing its translation into proteins and thus reducing the production of the mHTT protein [72]. However, the BBB poses a significant challenge as it hinders the efficient delivery of siRNA molecules [72]. Previous studies, such as Wang et al. [73], used invasive delivery methods of siRNAs in HD mice, such as intraventricular injections. FUS may provide a more precise and less invasive solution, enabling targeted delivery to critical brain regions.

A key investigation in this area was conducted by Burgess et al. [32], who demonstrated the efficacy of FUS in delivering siRNA to knock down mHTT gene expression in HD adult Wistar rats models. MRI confirmed the effective temporary disruption of the BBB, enabling cholesterol-conjugated siRNA that targets the HTT gene (cc-siRNA-HTT) to penetrate the targeted brain areas. Post-treatment analysis showed a significant, dose-dependent reduction in HTT mRNA levels in the striatum, with the optimal dose and maximum reduction observed being approximately 32%. Furthermore, a direct correlation was found between the extent of BBB disruption and the degree of HTT mRNA reduction, emphasizing the significance of precise control over the FUS parameters. This targeted delivery system showed specificity for the mHTT mRNA without significant off-target effects, enhancing safety and reducing the risk of systemic side effects.

Similar innovative approaches are being explored with adeno-associated virus (AAV) vectors to further enhance gene therapy for HD. AAV is particularly beneficial because it can deliver genetic material to both dividing and non-dividing cells and has a low immunogenicity profile, making it suitable for long-term gene expression. In HD, researchers are investigating AAV9 to deliver therapeutic genes aimed at mitigating the effects of the disease, which is characterized by progressive neurodegeneration [74]. However, delivering AAV across the BBB remains challenging, forcing researchers to use invasive direct brain injections as a method of delivery [66, 75, 76].

Building on these findings, Owusu-Yaw et al. [66] assessed the efficacy of FUS with AAV9 in gene transfer using the zQ175 mouse model of HD. This animal model carries a human mHTT gene and exhibits many key characteristics of HD pathology, including mHTT aggregation, increased neuroinflammation, and blood-brain barrier (BBB) breakdown at later disease stages, which align with human HD pathophysiology. Researchers conducted a study using single-stranded AAV9 encoding a Green Fluorescent Protein (GFP) reporter gene in wild-type and zQ175 HD model mice at different disease stages (2, 6, and 12 months). Their study showed improved AAV9 delivery and similar efficacy in wild-type and HD mice, though GFP expression was reduced in older zQ175 mice. It is crucial to highlight that even in older zQ175 mice, FUS did not increase astrocytosis, which is the proliferation of astrocytes in response to injury, suggesting safety [72].

While the reduced GFP expression in older mice suggests that age and disease progression may influence the efficacy of this method, the overall findings underscore the potential of FUS as a non-invasive, precise tool for gene therapy. Generally, these studies in oligonucleotide-based therapies and targeted delivery methods with the ability to selectively silence the mHTT while minimizing the off-target effects represent a significant leap forward in the treatment of HD. Advancements that enhance the efficiency of siRNA and AAV therapies across the BBB through developing novel nanocarriers and improved FUS techniques would be welcomed. Furthermore, it is crucial to assess the safety and efficacy of these oligonucleotide-based therapies in humans and try to strike a balance between reducing mHTT levels and preserving the essential functions of the normal HTT protein. Table 1 summarizes the previously discussed preclinical studies on FUS-mediated treatments in HD.

**Table 1:** Summary of the preclinical trials on FUS-mediated BBB opening in HD [32, 65, 66]

Study Name	Purpose	Therapeutic Agent	Animal model used	FUS Parameters	Target Area	Key Findings	Safety Profile
Owusu-Yaw et al. [66]	To evaluate the efficacy of FUS-mediated delivery of AAV9-based gene therapy in 2-month-old WT mice and the zQ175 HD mouse model at 2-, 6-, and 12-months.	AAV9-U6-miR10150-CBA-GFP vector	zQ175 HDmouse models	Frequency: 690 kHz Burst length: 10 ms Repetition frequency: 1 Hz Duration: 120 s Pressure: 0.34 MPa	Striatum and cortex	- FUS Treatment Impact: Improved AAV9 delivery for all mouse groups.- GFP Expression: Strong GFP expression in neurons and astrocytes.- Delivery Efficacy: Similar efficacy in all WT and HD groups, except for zQ175 12-month cohort.	Astrocytosis did not increase after FUS treatment, even within the zQ175 12-month group exhibiting higher baseline levels of GFAP¹ expression.
Burgess et al. [32]	To evaluate the non-invasive delivery of siRNA targeting mHTT to the right striatum using FUS and microbubbles, with the left striatum serving as the control.	siRNA-HTT	Wistar rats	Frequency: 558 kHz Estimated in situ pressure: 0.3 MPa Burst duration: 10 ms Pulse repetition frequency: 1 Hz Total exposure duration per sonication: 120 s Sonications: Conducted twice, with a one-hour interval between sessions Microbubble contrast agent: Definity^®^ (0.02 ml/kg) injected immediately before sonication	Striatum	- A significant 32% decrease in HTT expression following delivery of siRNA-HTT to the striatum with FUS.- The reduction in HTT DNA was dose-dependent, with higher siRNA-HTT concentrations.- The reduction in HTT with siRNA-HTT was more pronounced as the extent of BBB disruption increased.	No FUS-related adverse effects were reported. However, prolonged suppression of HTT may impair neuronal function, though a reduction of up to 30% appears to be tolerable.
Lin et al. [65]	To assess FUS-BBB opening for the delivery of a non-viral gene delivery system (LpDNA) containing both the pLuc-N3 and GDNF genes	GDNFp-LPs	R6/2 transgenic mouse models	Acoustic pressure: 0.33 MPa Frequency: 500 kHz Burst duration: 10 milliseconds Pulse repetition frequency (PRF): 1 Hz Duty cycle: 1% Total exposure time: 30 s on the contralateral hemisphere and 60 s on the ipsilateral hemisphere Microbubble (MB) dose: SonoVue^®^ at 0.1 mg/kg	Striatum and cortex	- GDNF was successfully expressed at targeted sites following FUS, with significant amplification of gene expression.- Enhanced transduction: 5-fold increase in intensity 2 days after FUS + LpDNA vs. LpDNA alone.- FUS-induced BBB opening led to higher concentrations of both drugs and genes at the target sites, demonstrating the stability and repeatability of the technique.	This study indicates the repeatability of FUS-mediated BBB opening, with no adverse events reported.

GFAP fluorescence intensity was quantified as a measure of the level of astrocyte activation.FUS: focused ultrasound; AAV9: adeno-associated virus serotype 9; WT: wild-type; HD: Huntington's disease; GFP: green fluorescent protein; GFAP: Glial fibrillary acidic protein; siRNA-HTT: small interfering RNA targeting Huntingtin; mHTT: mutant HTT; BBB: blood-brain barrier; GDNF: glial cell line-derived neurotrophic factor; LpDNA: liposomes containing plasmid DNA

## Discussion on Precision, Safety, and Efficacy of FUS

### Precision and Safety

Since the inception of FUS technology, its precision has been a focal point in research. Both spatial and temporal precisions are cornerstones in determining the applicability of various techniques. Studies dating back to the 1990s established that FUS could accurately target specific organs while sparing surrounding tissues, achieving a spatial precision of 250–300 microns, roughly the size of 10 hepatocytes [77]. A similar recent study on myocytes highlighted that the distance between affected and unaffected areas by FUS was inappreciable. This study showed that FUS can affect one half of a cell while preserving the other [78]. Additionally, combining FUS with MRI, which is the standard approach now, has markedly enhanced its spatial accuracy [79, 80] given the invaluable insights provided by the imaging regarding sonication parameters and target boundaries [81].

Since 2001, when FUS was first demonstrated to safely and effectively open BBB in animal models [49], its potential for human application has been increasingly recognized [82]. Through the fast-track translation of the emerging technique, its safety is now established in humans as well. The technique shows no long-term deficits in BBB function or structure, creating a temporary opening that typically lasts up to 24 h [56, 62–64, 83]. This provides a crucial window for drug administration, allowing therapeutic agents to reach the brain while ensuring the BBB returns to its normal state afterward. Moreover, there is repeated BBB opening with complete healing each time and no histological or physiological damage [84].

Clinical studies by Abrahao et al., Lipsman et al., Rezai et al., Mehta et al., and Gasca-Salas et al., have corroborated the safety of FUS in patients with neurodegenerative diseases such as AD, PD, and ALS, reporting no adverse effects such as intracranial hemorrhage, cognitive or neurological deficits, or physiological disruptions [56, 62–64, 83]. FUS is notable for its high spatial specificity and non-invasive nature, along with the fact that the energy needed to open the BBB is three times lower than that required to damage tissue. These unique features enhance the safety profile of FUS, especially in treating conditions like HD, where preserving brain integrity is paramount [85].

However, there are some pre-clinical trials on the long-term adverse events and safety of repeated FUS application that reported minimal side effects and this should be considered despite the safe application in the clinical trials of neurodegenerative diseases given the chronic course of HD. For instance, a study on sheep receiving weekly FUS-mediated BBB openings over six weeks found no abnormalities or behavioral changes in any experimental animals, though a single animal exposed to higher acoustic pressures (0.58 MPa) experienced minor, localized cerebral hemorrhage [86].

Additional research has explored vascular implications of FUS. One study observed transient blood supply reductions in regions with BBB disruption, suggesting FUS may impact vascular integrity in certain cases [87]. A transient hyperemic response in the unsonicated contralateral hemisphere also indicated a possible systemic response.

Moreover, FUS-induced BBB disruption has been associated with transient inflammatory responses. Kovacs et al. reported elevated levels of pro-inflammatory molecules and activation of microglia and astrocytes following FUS, raising concerns about cumulative inflammatory effects from repeated treatments [88]. McMahon and Hynynen further explored the acute inflammatory response following FUS-induced BBB permeability increase [89]. They found that the inflammatory response was dependent on MB dose, suggesting that optimizing MBs administration could help minimize inflammatory risks.

Clinical trials of FUS-mediated BBB opening in neurodegenerative diseases were supported by other preclinical studies that reported complete short- and long-term physical and functional safety profiles of its application in non-human primates such as rhesus macaques and others [90, 91]. However, careful monitoring and optimizing its parameters are recommended during its administration in human patients.

### Efficacy of FUS and Challenges of Translation

Preclinical trials on FUS-mediated BBB opening in HD models consistently demonstrate promising outcomes [32, 65, 66], including increased brain concentrations of therapeutic agents, along with significant reductions in mHTT mRNA levels and abnormal cytoplasmic HTT protein aggregates. These findings align with similar preclinical studies on other neurodegenerative diseases, including ALS and AD [92, 93], as well as brain tumors like glioma and glioblastoma [94, 95]. Furthermore, FUS has shown the ability to lower the required drug dosage needed for achieving the desired therapeutic effect, potentially minimizing side effects while maximizing efficacy [93].

Currently, no clinical trials have been completed, are active, or are recruiting patients to investigate the impact of FUS-based BBB disruption specifically in HD patients. The only clinical intersection between FUS and HD to date is a trial (NCT02252380) examining FUS-mediated ablation of targeted brain regions for managing movement disorders, including HD, through HIFUS [96].

Despite promising preclinical data, several limitations hinder the clinical translation of FUS in other neurodegenerative diseases. These challenges encompass limited external validity of clinical trials due to small sample sizes, the lack of a clearly defined threshold for effective BBB opening in humans, and a lack of conclusive findings on the impact of MB factors and sonication parameters on BBB permeability and various brain regions [64, 97].

Regarding HD, biological challenges are another type of challenge that might be faced. Since HD involves neuroinflammatory processes and MBs may be associated with transient inflammatory responses [89], it is essential to rigorously assess potential interactions between these inflammatory mechanisms before progressing to clinical trials. This is critical to ensure that FUS does not exacerbate the underlying pathology in HD patients. Further, the definite long-term consequences of BBB opening in humans is still unclear. Mehta et al., in their study on FUS-mediated BBB opening in AD patients, recommended long-term follow-up and post-mortem analyses of participants in FUS trials to gain a deeper understanding of the therapy’s prolonged impact [97]. Developing human-relevant in vitro models, such as those derived from induced pluripotent stem cells, may provide valuable insights into the specific responses of human BBB to FUS treatment in the context of HD. These models could help bridge the gap between animal studies and human clinical trials, accelerating the translation process while ensuring patient safety.

## In vitro BBB Models and FUS Application in HD

Human in vitro BBB models hold significant promise for translating preclinical findings regarding FUS applications in HD. These models typically consist of cells that replicate the structure of the human BBB, including the NVUs cultured on scaffolds that mimic the extracellular matrix [40].

These in vitro BBB models are derived from human-induced pluripotent stem cells (hiPSCs), which are reprogrammed [98] from somatic cells such as blood or skin cells using specific transcription factors [99, 100]. hiPSCs possess unlimited proliferative capacity and can be differentiated into mature cell types that closely resemble their in vivo counterparts. In the context of BBB models, hiPSCs can be engineered to carry genetic mutations associated with neurodegenerative diseases, thereby reflecting the pathological changes observed in the BBB [101–105]. Consequently, these models can replicate the altered microvascular environment characteristic of neurodegenerative disorders. Furthermore, researchers can develop personalized BBB models for individual patients since hiPSCs can be generated from a patient’s fibroblasts [22]. This capability has spurred investigations into drug delivery systems within the context of neurodegenerative diseases, aided by advancements in differentiation protocols and the ability to accurately reflect the BBB environment in vitro.

hiPSC-derived BBB models have been extensively utilized in FUS studies of neurodegenerative diseases other than HD [106–108] because they not only replicate in vivo drug permeability but also allow for precise control over experimental conditions. The ability to manipulate FUS parameters, accurate quantification of BBB opening, and smooth high-throughput drug screening offered by these models make them well-suited for FUS applications [109]. Additionally, in vitro BBB models allow for the exploration of sonication-associated effects at single-cell resolution, a level of detail that is not achievable in live human subjects [40]. Recent research has elucidated the parameters of FUS and MBs relevant to both human patients and BBB models of neurodegenerative diseases. A comprehensive comparison of these parameters across in vitro BBB models and clinical studies involving patients with AD, ALS, and PD is detailed in the review by Wasielewska et al., which draws on results from three clinical trials and a study by Oikari et al. focusing on a BBB in vitro model [40, 56, 62, 64, 110].

### Benefits of in vitro BBB Models for FUS in HD

Current research faces significant challenges in identifying primary BBB dysfunctions in HD and elucidating their underlying mechanisms through animal models and postmortem tissues [111]. In addition, understanding how FUS specifically exerts its effects is crucial for clinical translation, particularly given the complex pathophysiology and changes in brain microvascularity associated with HD [111–113]. Personalized hiPSC-derived BBB models provide a promising solution for these two issues by reflecting the unique conditions of HD patients’ BBBs [114]. Studies using HD in vitro BBB models have begun to investigate the impact of CAG repeat expansions on BBB properties, including transendothelial electrical resistance, receptor-mediated transcytosis, and responses to both physiological and pathological stressors, such as oxidative and osmotic stress [111, 114]. Further studies have evaluated the permeability of HD-related drugs (e.g., arginine, verapamil, and vinblastine) through these models, contributing to our understanding of drug transport and BBB resilience under HD conditions [112]. These advanced in vitro models can mitigate interspecies differences and bridge the gap between animal findings and clinical trials by providing individualized parameters for safer and more effective treatment strategies.

Despite the insights gained from related neurodegenerative disease studies, the literature currently lacks research applying FUS in an HD in vitro BBB model. In contrast, over ten clinical trials have explored BBB opening through FUS in AD patients, where ongoing in vitro studies aim to address the limitations in these clinical trials. Most existing clinical trials have primarily focused on the safety and efficacy of FUS in BBB opening without assessing drug delivery efficacy due to a limited understanding of FUS mechanisms and their interactions with brain vasculature. For example, a recent study on an AD patient-derived in vitro BBB model demonstrated that FUS could enhance the permeability of a 5 kDa cargo molecule and revealed clinically relevant differences in post-treatment recovery durations between disease genotypes, as well as insights into how endothelial cells interact with pericytes and astrocytes that may influence the BBB’s response to therapeutic ultrasound [110]. These findings highlight the potential benefits of patient-specific BBB models in HD research, enhancing our understanding of FUS-induced permeability changes and promoting targeted therapeutic strategies for HD.

By leveraging insights from in vitro BBB models, researchers can refine FUS protocols and drug delivery strategies tailored to individual patients, ultimately enhancing patient outcomes. These models may address limitations encountered in animal trials of FUS for HD by exploring the properties of BBB dysfunction associated with HD and optimizing parameters to ensure human tolerability for future clinical trials.

## Conclusion

FUS is a noninvasive technology that can cause a temporary opening of the BBB, allowing therapeutic molecules to enter the brain, and is particularly promising. This innovative method significantly increases the bioavailability of neuroprotective agents for HD improving therapeutic outcomes in animal models. Although preclinical studies have demonstrated encouraging results, the clinical application of FUS in HD is still in its infancy. However, as clinical trials begin to explore the safety and effectiveness of FUS in humans with other neurodegenerative diseases, this will bring us closer to transforming these discoveries into practical treatments of neurodegenerative diseases, including HD. This progress offers hope for significantly changing the course of the disease and improving the lives of those affected.

## Data Availability

No datasets were generated or analysed during the current study.
